# Virus-independent and common transcriptome responses of leafhopper vectors feeding on maize infected with semi-persistently and persistent propagatively transmitted viruses

**DOI:** 10.1186/1471-2164-15-133

**Published:** 2014-02-14

**Authors:** Bryan J Cassone, Saranga Wijeratne, Andrew P Michel, Lucy R Stewart, Yuting Chen, Pearlly Yan, Margaret G Redinbaugh

**Affiliations:** 1 USDA, ARS Corn, Soybean and Wheat Quality Research Unit, Wooster, OH 44691, USA; 2 Molecular and Cellular Imaging Center, Ohio Agriculture Research and Development Center (OARDC), Wooster, OH 44691, USA; 3 Department of Entomology, Ohio State University, OARDC, Wooster, OH 44691, USA; 4 Department of Plant Pathology, Ohio State University, OARDC, Wooster, OH 44691, USA; 5 Human Cancer Genetics Program, Ohio State University, Columbus, OH 43210, USA; 6 Department of Molecular Virology, Immunology and Medical Genetics, School of Biomedical Science, Ohio State University, Columbus, OH 43210, USA

**Keywords:** Gene expression, Leafhopper, Nucleorhabdovirus, Waikavirus, Viral transmission pathogen response, Innate immunity

## Abstract

**Background:**

Insects are the most important epidemiological factors for plant virus disease spread, with >75% of viruses being dependent on insects for transmission to new hosts. The black-faced leafhopper (*Graminella nigrifrons* Forbes) transmits two viruses that use different strategies for transmission: *Maize chlorotic dwarf virus* (MCDV) which is semi-persistently transmitted and *Maize fine streak virus* (MFSV) which is persistently and propagatively transmitted. To date, little is known regarding the molecular and cellular mechanisms in insects that regulate the process and efficiency of transmission, or how these mechanisms differ based on virus transmission strategy.

**Results:**

RNA-Seq was used to examine transcript changes in leafhoppers after feeding on MCDV-infected, MFSV-infected and healthy maize for 4 h and 7 d. After sequencing cDNA libraries constructed from whole individuals using Illumina next generation sequencing, the Rnnotator pipeline in Galaxy was used to reassemble the *G. nigrifrons* transcriptome. Using differential expression analyses, we identified significant changes in transcript abundance in *G. nigrifrons*. In particular, transcripts implicated in the innate immune response and energy production were more highly expressed in insects fed on virus-infected maize. Leafhoppers fed on MFSV-infected maize also showed an induction of transcripts involved in hemocoel and cell-membrane linked immune responses within four hours of feeding. Patterns of transcript expression were validated for a subset of transcripts by quantitative real-time reverse transcription polymerase chain reaction using RNA samples collected from insects fed on healthy or virus-infected maize for between a 4 h and seven week period.

**Conclusions:**

We expected, and found, changes in transcript expression in *G. nigrifrons* feeding of maize infected with a virus (MFSV) that also infects the leafhopper, including induction of immune responses in the hemocoel and at the cell membrane. The significant induction of the innate immune system in *G. nigrifrons* fed on a foregut-borne virus (MCDV) that does not infect leafhoppers was less expected. The changes in transcript accumulation that occur independent of the mode of pathogen transmission could be key for identifying insect factors that disrupt vector-mediated plant virus transmission.

## Background

Among the most difficult plant diseases to control are those transmitted by insect vectors. Pathogens and their vectors have co-evolutionary histories that are intricately intertwined with their ecologies, environments and genetic interactions. While much progress has been made towards understanding the interactions among vectors and pathogens required for transmission of human and animal diseases [1,2], much less is known about the factors that govern the transmission of insect mediated plant diseases. A detailed understanding of the genetic and molecular basis of insect-virus-plant interactions will provide more accurate predictions of how vector populations are likely to respond to control measures, and may uncover novel and specific molecular targets for disease control.

Modes of plant virus transmission by insects fall into four categories: non-persistent, semi-persistent, persistent-circulative, and persistent-propagative. Non-persistently-transmitted and semi-persistently-transmitted viruses do not breach the gut barrier in the insect vector; rather they are retained in the insect stylet or foregut prior to transmission. Because of their location outside of insect cells and the few barriers they must cross prior to introduction into a new plant host, these viruses can be transmitted within seconds to hours after acquisition. Most viruses transmitted in a non-persistent manner are transmitted by aphids; aphids, leafhoppers and whiteflies transmit semi-persistently-transmitted viruses [3,4]. Persistently transmitted viruses circulate within the insect vector before transmission, usually moving through the gut lumen into the hemolymph or other tissues and finally invading the salivary glands prior to dissemination to new host plants during insect feeding [5]. Persistently transmitted viruses are divided into two categories: circulative viruses that are able to cross the gut barriers and circulate in the insect hemolymph, but do not replicate in the insect vector; and, propagative viruses that circulate in and also replicate in the vector. Because these viruses must cross several molecular and physical barriers in the insect and require a latency period between acquisition and transmission, their transmission requires days to weeks. Persistently and propagatively transmitted viruses are transmitted by thrips, leafhoppers, planthoppers and aphids, whereas viruses transmitted in a persistent circulative manner are primarily transmitted by whiteflies, aphids and leafhoppers [3]. Because non- and semi-persistently transmitted viruses interact only briefly with the vector, and do not cross vector membranes, we hypothesized that the genetic and molecular responses of the vector to these viruses are likely to be substantially different and perhaps less diverse than those of the vector to persistently transmitted plant viruses.

The black-faced leafhopper, *Graminella nigrifrons* (Forbes) is found in the eastern U.S., feeding on a wide range of grasses and cereal crops [6,7]. It is the principal vector of two viruses that infect maize (*Zea mays* L.): *Maize chlorotic dwarf virus* (MCDV) and *Maize fine streak virus* (MFSV). MCDV is a member of the genus *Waikavirus* that was first detected in maize in Ohio and is prevalent in the southeastern U.S. [8,9]. MFSV is an emerging *Nucleorhabdovirus*, first reported in maize in Georgia, U.S. [10]. While MCDV is semi-persistently transmitted, with acquisition and inoculation to host plants occurring in as little as four hours [11]. MFSV is persistently and propagatively transmitted, requiring replication in the insect. Acquisition and inoculation periods totaling at least three weeks are required before they are able to transmit virus to new plant hosts [12].

Virus-induced biochemical, cellular, molecular, and physiological changes have been well documented in infected host plants [13], but the responses of insect vectors to viruses are far less well characterized. Viruses can influence their insect hosts either directly through deleterious effects of virus infection, or indirectly by virus-induced changes in host plant phloem components (e.g. nutritional substances, toxins, etc.). Even in cases where the vector becomes infected, effects of the virus in the insect tend to be less severe than in the host plant [5]. Insects use a variety of defense mechanisms to combat pathogen infection [14]. These responses include the synthesis and secretion of antimicrobial peptides and degrading enzymes, phagocytosis, cell apoptosis, and cell sloughing. Assessing changes in transcript abundance in response to pathogen exposure was critical for identifying contributors to the molecular and cellular defense responses in insect vectors of animal disease, and also provided insight into mechanisms associated with pathogen defense [1]. Transcript accumulation is intimately tied to transcript function, and can be used to infer an organism’s functional responses under different conditions. In insects, transcriptional induction or repression is a key mechanism in regulating innate immunity [1]. However, gene expression studies investigating plant virus – insect vector interactions remain limited, with only a few studies characterizing global transcription profiles of insect vectors fed on virus-infected plants [15-18]. Some transcriptional data are also available for insect vectors of animal-infecting rhabdoviruses [19-21] and plant-infecting rhabdoviruses [22]. To date, no study has investigated transcriptional responses to non-persistently transmitted viruses or to differences in vector responses based of the modes of virus transmission.

The recent sequencing and assembly of the *G. nigrifrons* transcriptome [23] provides a valuable tool for exploring transcriptional changes in an important vector of plant pathogens. In this study, we used next generation sequencing to reassemble the transcriptome and examine changes in transcript accumulation between *G. nigrifrons* fed on MCDV-infected, MFSV-infected, and healthy maize for 4 h and 7 d. MCDV and MFSV were chosen to examine divergent responses based on the mode of transmission, and multiple time points were selected to characterize both the immediate and gradual impacts of feeding on virus-infected plants. Transcriptome analysis revealed a substantial, shared induction of core immunity and energy metabolism genes in the response to feeding on virus-infected plants. Feeding on MFSV-infected plants elicited additional responses, which included increased transcription of hemocoel and cell-membrane associated immune processes. Patterns of transcript expression were validated on a subset of transcripts using quantitative real-time reverse transcription polymerase chain reaction at 4 h, 7 d, and six additional time points across a four week time period.

## Results and discussion

### Sequence assembly

One cDNA library consisting of 24 pooled samples was sequenced, which generated approximately 446 million raw 100 bp paired end reads. After demultiplexing, trimming poor quality reads, adapters and poly(A) sequences, and removing duplicate reads, 6.8 to 20 million non-redundant reads per sample were obtained. *De novo* assembly of the *G. nigrifrons* transcriptome was carried out using an automated pipeline, and combined with the previously assembled EST database [23] resulting in 38,113 *G. nigrifrons* contigs of ≥200 bp (1,160 bp mean length). Thirteen contigs identified >20 kb were atypical of eukaryotic transcripts and probably represent long non-coding RNAs [24]. After mapping to the Swiss-Prot and nr databases (*E*-value <10^-6^), 576 contigs were removed from the dataset because they were of maize, bacterial or virus origin. The remaining 37,537 contigs form the dataset for subsequent analyses and are hereinafter referred to as ‘transcripts’. The contig sequences have been deposited in the NCBI Transcriptome Shotgun Assembly archive under the accession number GAQX00000000.

### Functional annotation and pair-wise comparisons with other invertebrate species

Approximately 42% (n = 15,659) of the transcripts had a significant hit to the Swiss-Prot database (*E*-value <10^-6^). Of the 1,516 most highly conserved transcripts (*E*-value <10^-180^), one-third (n = 515) had greatest amino acid sequence identity to a *D. melanogaster* ortholog (Additional file 1: Table S1). BLAST2GO was used to examine the hierarchical associations of *G. nigrifrons* transcripts by assigning gene (GO) and enzyme (EC) ontologies during the mapping and annotation steps. GO and/or EC terms in the nr database mapped to 7,349 transcripts and 4,855 could be annotated. Of the annotated transcripts, 3,279 were associated with a biological process, 3,998 with a molecular function, and 2,731 with a cellular component (Additional file 2: Table S2).

**Figure 1:**
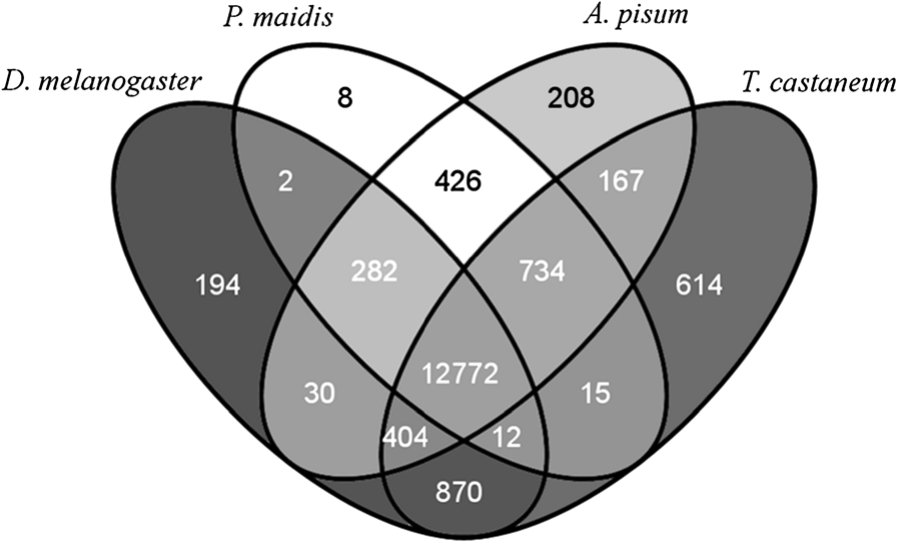
**Venn diagram showing tBLASTx (*E*-value <10**^**-10**^**) pair-wise ortholog matches of *Graminella nigrifrons *** **to three invertebrate species with characterized transcriptomes (*D. melanogaster*: 19,233 transcripts; *A. pisum*: 37,994 transcripts; *T. castaneum*: 14,366 transcripts) and one species with a dbEST database (*P. maidis*: 10,636 ESTs).**

Pair-wise sequence comparisons were carried out between the *G. nigrifrons* transcriptome and those of seven well-characterized invertebrates and one insect dbEST collection. Pair-wise matches between *G. nigrifrons* and four insect species indicated that roughly 46% (n = 17,174) of transcripts had a significant match to at least one of the insect databases (*E*-value <10^-10^) (Figure 1). *G. nigrifons* transcripts had the fewest matches to *C. elegans* transcripts (n = 10,540) and the most matches to *T. castaneum* (n = 15,566). The number of matches ranged from 14,233 to 15,301 for the other seven insect comparisons (*D. melanogaster*: 14,547; *An. gambiae*: 14,736; *P. maidis*: 14,233; *A. mellifera*: 15,301; *N. vitripennis*: 15,214; *A. pisum*: 15,002).

For the subset of *G. nigrifrons* transcripts with an ortholog match (n = 17,174), 620 had a unique match to only one species. Taking into account the relative variability in transcriptome/dbEST sizes, *T. castaneum* had the largest proportion of unique matches (43%, 195). The hemipteran, *A. pisum*, accounted for 11% of the unique matches (n = 129), whereas *C. elegans* accounted for only 2.8% (n = 30). The proportion of unique matches for the dipterans and hymenopterans ranged between 5.4% and 15%: *An. gambiae*: 11% (n = 51); *D. melanogaster*: 5.4% (n = 33); *N. vitripennis*: 9.8% (n = 84); *A. mellifera*: 15% (n = 91). *G. nigrifrons* had the smallest proportion of unique transcript matches with the planthopper *P maidis* at 2.2% (n = 7) despite its being the most closely related species. This result was surprising and may be because the *P maidis* library was derived only from gut tissue, and also examined at different time points and developmental stages. Fewer assembled *G. nigrifrons* transcripts were identified in this study than in the initial 2012 assembly of Chen et al. (37,548 vs. 38,240), with <1% (n = 246) of the transcripts from Chen et al. not matching a transcript in the current assembly (BLASTn, *E*-value <10^-10^). The majority of the unmatched transcripts (n = 144) could not be assigned a function using the non-redundant databases (tBLASTx, *E*-value <10^-10^). More of the transcripts in this study had significant hits to the Swiss-Prot database (15,659 vs. 13,036), mapped to GO and/or EC terms (7,349 vs. 4,488) or matched to an insect database (17,174 vs. 14,259). It is likely that the increased read coverage (446 million vs. 63 million), longer read sizes (100 bp vs. 76 bp), additional time points, different treatments, and/or improved preprocessing and assembly techniques contributed to a more complete and accurate construction of the *G. nigrifrons* transcriptome.

### Treatment and time dependent differences in transcript expression profiles

Transcript expression levels were computed for each treatment/time point sample independently as the unique (i.e. unambiguous) reads per kilobase per million reads (RPKM). Treatment was defined as leafhoppers fed on either MCDV-infected, MFSV-infected or healthy maize. Time was defined as the duration of feeding, either 4 h or 7 d. Quality control parameters indicated the RPKM file contained samples that were normally distributed and homogenous. The total number of unique reads per sample used for RPKM analysis ranged from 1.6 million to 4.6 million. Reproducibility between replicate treatment/time combinations was high, with average R^2^ values of 0.89 (range between 0.86 and 0.94).

A linear mixed model ANOVA was used (*P* <0.05) to identify transcriptome differences as a function of treatment, time, and their interaction, under the hypothesis the expression profile would differ significantly between time points. Both time and the interaction factor had a significant influence on overall transcript expression, while treatment was not significant at *P* <0.08 (Table 1). More than half (n = 19,952) of the transcripts accumulated to significantly different levels with respect to one or more factors. Because time and its interaction with treatment were significant factors in the model, subsequent analyses separated the data by sampling times to examine the effects of treatment at each time point.

**Table 1:** **The effects of treatment, time, and their interaction term on transcript levels in *G. nigrifrons *** **virgin females**^**1**^

**Factor(s)**	**df**^**2**^	**SS**	**MS**	***F***	***P-*value**
Treatment	2	31130	15564	2.6	0.078
Time	1	82470	82741	13.5	2.38E-04
Time x Treatment	2	39970	19985	3.3	0.038

^1^Analysis of variance for abundance of transcripts.

^2^df, degrees of freedom; SS, sum of squares; MS, mean square; *F*, F test statistic.

### Transcriptome variation in *G. nigrifrons* in response to feeding on virus infected maize

To determine if feeding on virus-infected maize elicited changes in *G. nigrifrons* that differed from those fed on healthy maize, the samples derived from leafhoppers fed on MCDV- and MFSV-infected plants were taken collectively for each time point, and Bayes-moderated *t*-tests (*P* <0.05) were carried out to identify common differentially expressed transcripts. At 4 h, 2,156 differentially expressed transcripts were identified, with the majority (n = 1,310) being up-regulated in leafhoppers fed on virus-infected maize. Fewer differentially expressed transcripts were identified at 7 d (n = 1,688), and a greater proportion of these were down-regulated (55%). Interestingly, only 93 transcripts (2.5%) were differentially expressed at both time points (Additional file 3: Table S3). The majority of these shared transcripts (n = 57) were not expressed in the same direction, i.e., they were induced at 4 h and repressed at 7 d or vice versa, further indicating change in the response of vectors feeding on infected plants over time. The vast majority of differentially expressed transcripts at both time points (4 h: 2,008; 7 d: 1,522) were also identified using the edgeR package in Bioconductor, indicating good correlation between the statistical methods. Among the 25 top-ranked transcripts with the greatest up-or down-regulation at each time point (determined by ranking RPKM-changes between leafhoppers fed on virus-infected and healthy plants), the majority (83%) could not be annotated using the nr database (Additional file 4: Table S4).

**Figure 2:**
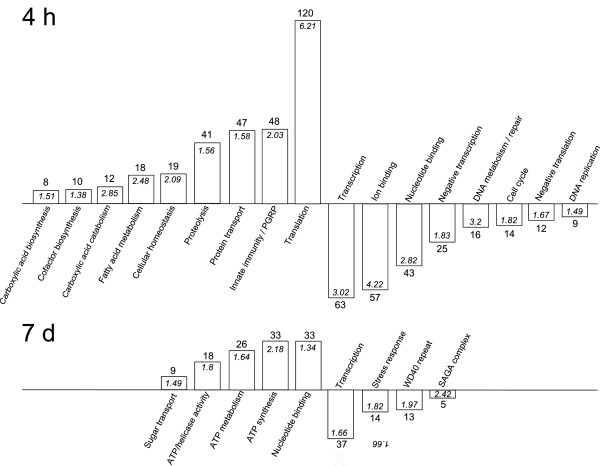
**Functional clustering of *Graminella nigrifrons *transcripts in response to feeding on virus-infected (MCDV and MFSV combined) and healthy maize. ***G. nigrifrons* transcripts with *Drosophila* orthologs were identified and clusters of functionally related transcripts were identified using DAVID (30). The numbers of significant transcripts in each cluster are indicated above the columns, and DAVID enrichment scores are displayed inside each column.

*G. nigrifrons* transcriptome annotation is in its earliest stages; therefore, we did not attempt formal quantitative functional analysis of differentially expressed transcripts. Instead, we selected an exploratory approach that relied on ortholog prediction using the *Drosophila* transcriptome. The DAVID annotation clustering module [25,26] set at medium stringency (default level) was used to classify differentially expressed *G. nigrifrons* transcripts into functional groups. Transcripts were first assigned their ortholog *Drosophila* ID (where possible) then partitioned into four lists: up- or down-regulated in leafhoppers fed virus-infected maize at 4 h or 7 d. Since only ~40% of *G. nigrifrons* transcripts had a *Drosophila* ortholog, this analysis only identified some of the processes affected by the treatments. However, the approach removed partial duplicates and variants of candidate transcripts that may not have been detected during the assembly, thus limiting the potential for false overrepresentations. Four to nine functional annotation clusters containing ≥5 transcripts that were more than 2-RPKM up- or down-regulation relative to the healthy control were identified in each of the four treatment/time point comparisons (Figure 2).

### Up-regulation of immunity transcripts within hours of feeding on virus-infected plants

*G. nigrifrons* fed on virus-infected plants had large and rapid (within 4 h) induction of transcripts that encode components of innate immunity pathways and the active homeostatic processes that regulate them (Figure 2A). The response was transient, as levels of most of these transcripts were similar in insects fed on virus-infected and healthy plants for seven days. Based on *Drosophila* ortholog prediction, immune response transcripts that were up-regulated included several members of the TOLL pathway (FBtr0-074275, 074275, 078240, 308648), peroxidases (FBtr00-72983, 73066), a superoxide dismutase (FBtr0076229), a scavenger receptor (FBtr0304741) and a clip-domian serine protease (FBtr0086060). Notably, transcripts encoding a subset of peptidoglycan recognition proteins (FBtr0-073509, 075320, 076439, 088708, 088709, 089490, 300291, 306099, 307978), which function as pattern-recognition and effector molecules in innate immunity [27], accumulated to high levels. Other groups of up-regulated transcripts included those encoding enzymes that function in proteolysis, especially the ubiquitin proteasome pathway, which was shown to be important for insect defense against pathogens [28]. The shared rapid response also included accumulation of transcripts related to protein and energy metabolism, including those associated with translation and the fatty acid and carboxylic acid metabolic pathways. The roles of these pathways in energy production may be important to compensate for the increased metabolic requirements of an elevated immune defense response [29].

Concurrent with the elevated immune response at 4 h after feeding on virus-infected plants, transcripts associated with regular cellular activity were suppressed (Figure 2A). In particular, transcripts involved in processes related to DNA replication and the cell cycle accumulated at lower levels in *G nigrifrons* fed on virus-infected plants. Virus suppression of host cell replication processes may represent a counter-defense strategy employed by viruses to promote their own replication [30]. Alternatively, the suppression could be the result of the added metabolic burden associated with the heightened immune response. A similar inhibition of basic cellular functions coinciding with increased energy generation has been identified in the transcriptional responses in insects exposed to other stresses, such as heat shock [31,32].

While the shared immune response was not detected after seven days, the up- and down-regulation transcripts with putative basic cellular functions (i.e., DNA replication) and increased energy production persisted (Figure 2B). This result suggests that ATP-requiring processes related to the virus response continue to occur at 7 d. While these data suggest a diminishing innate immune response with time, it is possible that the early stage annotation of the *G. nigrifrons* transcriptome prevented detection of immune responsive transcripts at the later time point.

### Common responses to feeding on virus-infected plants likely reflect some combination of plant defense responses and direct virus effects

The substantial, shared and rapid transcriptional responses in *G. nigrifrons* fed on both MFSV- and MCDV-infected maize were unexpected given the substantial differences in the ways these two viruses are transmitted, and could indicate that the leafhoppers are responding not only to the virus *per se*, but also to similar changes that occur in maize infected with either virus. Virus infection causes biochemical, cellular, molecular, and physiological changes in host plants [13,33,34], some of which increase plant attractiveness and preference to vectors [35-40]. However the transcript expression patterns observed were consistent with recent studies of the white-backed planthopper *Sogatella furcifera* and the whitefly *Bemisia tabaci* exposed to persistently transmitted viruses, which showed changes in accumulation of innate immunity transcripts [16,17]. In these studies, the insect was allowed to feed on healthy plants for several days after virus exposure to allow adequate time to diminish potential differential effects of the infected plant (e.g. volatiles, defenses responses), a strategy not possible in our study due to the short retention time of MCDV in *G. nigrifrons*. Future expression studies to compare leafhoppers fed on purified virus in artificial diets independent of the host plant or directly injected with virus may better resolve this for certain transcripts, but the process of feeding insects on artificial media containing virus also introduces a number of new variables that could potentially initiate transcript expression profiles unrelated to the vector – virus interaction (see [16]).

### Differential response of *G. nigrifrons* to feeding on MFSV- and MCDV-infected maize

**Figure 3:**
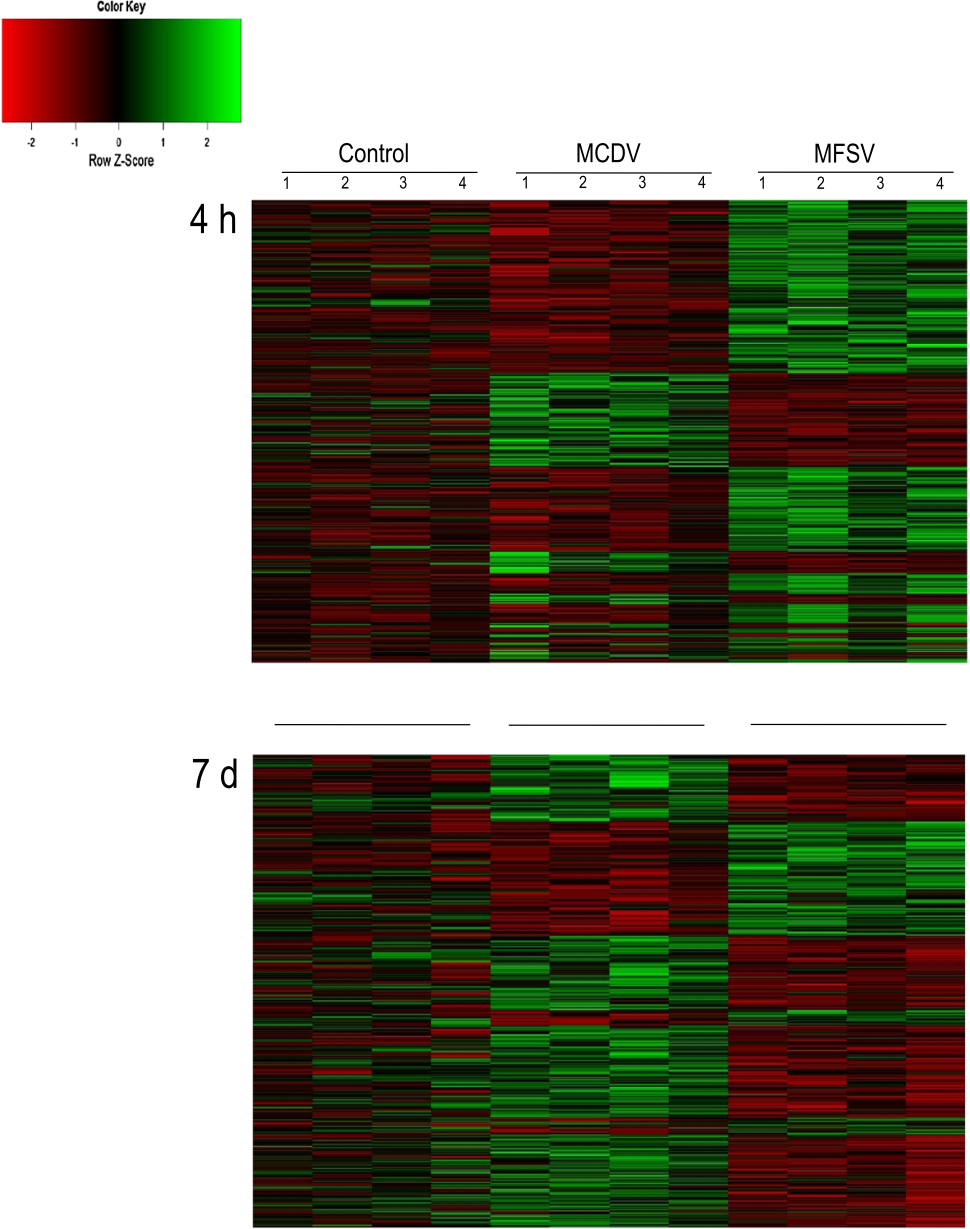
**Transcriptional profiles of the 1,046 and 536** ***G. nigrifrons *transcripts with a significant treatment effect at 4 h and 7 d, respectively.** Both analyses are based on one-way ANOVA with *P*-level significance at 0.05 and RPKM change >2. Each row represents an individual transcript; each column labeled 1–4 represents a replicate sample that was fed on healthy (control) or virus-infected (MCDV or MFSV) maize. For each transcript (row), the relative expression level in each sample (column) is represented by a color that reflects its row z-score (shown in the grayscale key), calculated by subtracting the mean expression value for the row from individual sample values and dividing by the standard deviation of the row.

Although feeding on MCDV- and MFSV-infected maize elicited a common differential accumulation of transcripts relative to those fed on healthy maize, virus-specific changes in transcript accumulation were also possible. One-way ANOVA was carried out to identify transcripts with a significant treatment effect at 4 h and 7 d. A total of 1,046 and 536 transcripts were differentially expressed with a minimum 2-RPKM-change difference between treatments at 4 h and 7 d, respectively. For the 4 h treatment, a heat map of differentially expressed transcripts indicated that the majority of these transcripts were differentially expressed in insects fed on MFSV-infected plants (n = 757), and that 84% of these were up-regulated (Figure 3A). At 7 d, the number of differentially expressed transcripts was similar in *G. nigrifrons* fed on MCDV- and MFSV-infected plants (Figure 3B). However, 66% of transcripts were down-regulated in insects fed on MFSV-infected maize, whereas 76% were up-regulated for insects fed on MCDV-infected plants. For 286 of 1582 transcripts, the response was opposite in the two treatments. Specifically, 29% and 19% of transcripts up-regulated in *G. nigrifrons* fed on MCDV- and MFSV-infected maize, respectively, were down-regulated relative to the control in the alternate virus treatment.

**Figure 4:**
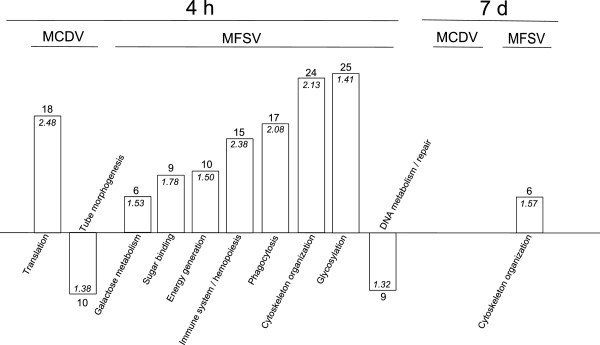
**Functional annotation clusters of *Graminella nigrifrons *transcripts in response to feeding on MCDV-infected, MFSV-infected, or healthy maize. ***Drosophila* orthologs were identified and clusters of functionally related transcripts were identified using DAVID [41]. The numbers of significant transcripts in each cluster are indicated above the columns, and DAVID enrichment scores are displayed inside each column.

To determine whether this differential transcript expression was associated with specific biological responses, the identified transcripts were divided into four lists (up- or down-regulated in *G. nigrifrons* fed on MCDV- or MFSV-infected maize) and functional annotation was done using DAVID. Significant enrichment of annotation clusters was found for three of four lists, with one and seven clusters identified as containing significant up- or down-regulation relative to the alternative treatment and healthy control (Figure 4).

### Additional immune-linked responses elicited by *G. nigrifrons* fed on MFSV-infected plants

The rapidly up-regulated transcripts in *G. nigrifrons* fed on MFSV-infected plants were largely associated with energy metabolism and hemocoel or cell membrane-associated defense responses, such as cytoskeleton organization, hemopoiesis, glycosylation, and phagocytosis/endocytosis. Glycosylation plays a key role in the immune response as a sugar-mediated mechanism in cell-cell adhesion for the detection of pathogen carbohydrate moieties [42]. Other differentially expressed transcripts encode components of the phagocytic process, whereby cells ingest and eventually destroy foreign particles, including pathogens [43]. Possible immune-response associated transcripts involved in hemopoiesis [44] and cytoskeleton organization [2] were also up-regulated. Cytoskeleton-dependent intracellular transport is a common strategy for intracellular virus transport [45-48]. Transcripts associated with cytoskeleton organization were also up-regulated at 7 d, and this was the only group with significant enrichment at 7 d.

Because persistently-propagatively transmitted viruses encounter multiple tissue and membrane barriers in the path from the alimentary canal to the salivary glands, feeding on MFSV-infected maize for up to 7 d did not afford leafhoppers a long enough latency period to acquire the virus [12]. However, the added immune responses may be induced by attempts of MFSV to breach the vector gut barriers and/or entry of the virus into the hemolymph of the vector. In contrast, semi-persistently transmitted viruses like MCDV are largely retained in the foregut and do not cross membranes. Our results lead to a working model in which exposure of *G. nigrifrons* to MFSV elicits greater immune responses than exposure to MCDV. The greater response to MFSV may be required to limit the replication and circulation of the virus in the vector, thereby reducing potential pathological effects on vector development, fertility, and fecundity. However, for *G. nigrifrons* feeding on MCDV-infected plants, any advantages associated with mounting additional immune responses could be outweighed by their metabolic cost, depleting energy reserves that could leave individual leafhoppers at a competitive disadvantage [49,50].

### RT-qPCR validation and expression profiling of differentially expressed transcripts

Differential expression of four transcripts was verified using RT-qPCR. The transcripts were randomly selected from the subset of transcripts that were identified by RNASeq as up-regulated in *G. nigrifrons* fed on virus-infected maize for 4 h, and for which a *Drosophila* ortholog was predicted. Primer pairs for the selected transcripts were developed such that primer pair PCR efficiencies (*E*-values) were between 1.9 and 2.15 (Additional file 5: Table S5). All four transcripts were confirmed to be significantly up-regulated in both the MFSV and MCDV treatments relative to the control treatment, with similar magnitudes of expression between the different quantification methods (Spearman’s coefficient *rs* = 0.94; *P* < 0.001).

**Figure 5:**
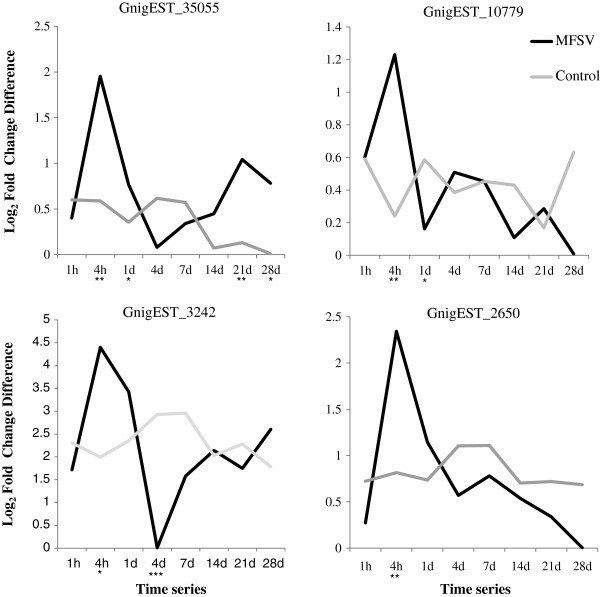
**Transcription profiles of GnigEST-35055, 10779, 3242, and 2660 for newly emerged adult *G. nigrifrons *females fed on MFSV-infected and healthy (control) maize at eight time points over a four-week window.** Relative log_2_-fold gene expression values are represented on the y-axis, and the time points are represented on the x-axis. Significant expression differences between treatments at each time point are denoted below the x-axis. *P*-values are indicated as *, *P* < 0.05; **, *P* < 0.01; ***, *P* < 0.001.

Accumulation of these four transcripts was also examined for the MFSV and control treatments at six additional time points across a four week window (Figure 5). For all four transcripts, the MFSV treatment exhibited a more heterogeneous pattern of expression across the time series, with highest transcript accumulation at 4 h and no discernible time points of peak expression for the control treatment. The patterns of expression were consistent among samples of the same treatment, with considerable decline in transcript accumulation in MFSV treatment after feeding on virus infected maize for 4 h. Taken collectively; all four transcripts appear induced in leafhoppers fed on MFSV-infected maize but not on healthy maize. As suggested by next generation sequencing analysis, the response was fleeting, as transcript accumulation returned to pre-feeding levels within 1 to 4 d. These results indicate that the transcriptome profiles of *G. nigrifrons* fed on virus-infected tissue are not consistent in accumulation but rather change in composition throughout the feeding/infection cycle.

## Conclusions

The most economically significant insect vectors of plant viruses are restricted to a few hemipteran families, including leafhoppers (Cicadellidae) [51]. Comparison of the responses of the leafhopper, *G. nigrifrons*, to feeding on maize infected with two different viruses uncovered similarities as well as clear differences in the transcriptional responses in the vector that may provide some mechanistic insight. Feeding on MCDV- and MFSV-infected maize for 4 h elicited a substantial and shared temporary induction of transcripts involved in innate immune response and energy production. Insects fed on MFSV-infected maize also had increased hemocoel and cell-membrane linked immune responses compared with *G. nigrifrons* after 4 h of feeding on MCDV-infected maize. The response did not persist, as the expression of most transcripts returned to pre-exposure levels within seven days. Identifying transcriptional changes in the vector that were independent of virus transmission mode was surprising, and may help facilitate the development of control measures aimed at disrupting vector competence. Moreover, emerging high throughout techniques to assay gene function may be undertaken to identify the transcriptional changes influencing *G. nigrifrons* virus transmission capability. Further studies are needed to better characterize the functional roles and interactions among differentially expressed transcripts, to evaluate expression patterns over time, and to determine their impact on fitness in natural populations.

## Methods

### Leafhopper colony and virus maintenance

Experiments were conducted using a laboratory colony of *G. nigrifrons* established from multiple, ongoing field collections taken near Wooster, OH since the early 1980s (40.8050° N, 81.9353° W). Leafhoppers were maintained on maize ‘Early Sunglow’ (Schlessman Seed Co., Milan, OH) seedlings placed in 15 cm x 7.5 cm x 15 cm cages in growth chambers with light–dark cycles of 28°C for 16 h and 25°C for 8 h.

MFSV and MCDV (MCDV-S) isolates [10,52] were maintained in the sweetcorn hybrid ‘Spirit’ by serial vascular puncture inoculation [53]. Infection efficiency for these experiments ranged between 40 – 75% for MFSV and 60 – 85% for MCDV based on symptom development in inoculated seedlings. Virus presence in experimental plants was verified using enzyme-linked immunosorbent assays as previously described [12].

### *G. nigrifrons* fed on MCDV- and MFSV

Each replication consisted of three treatments (feeding on MFSV-infected, MCDV-infected, or healthy maize) with two time points (4 h or 7 d) per treatment. Two hundred and forty newly emerged virgin females (≤24 h after molt from 5^th^ instar nymph stage) were randomly chosen in roughly equal numbers from three maintenance cages, and starved for 3 h. For each treatment/time combination, 40 females were divided evenly between two 0.1 m^3^ cages each containing one 3.5 – 4 wk old MFSV-infected, MCDV-infected, or healthy (control) maize plant. After 4 h or 7 d, the insects from each cage were collected, frozen in liquid nitrogen, and stored at −80°C until RNA isolation. While the acquisition access period (AAP) is likely a little shorter than the time point collected, only individuals in the process of feeding were collected. Each treatment/time point was replicated four times using leafhoppers from different cohorts (24 samples total).

### RNA isolation and cDNA library preparation

Total RNA was extracted from pools of 25 leafhoppers using the RNeasy Mini Kit (QIAGEN, Germantown, MD). RNA quantity and quality were assessed using the Experion Automated Electrophoresis System (Bio-Rad, Hercules, CA). Only samples with RNA Quality Indicator values of 10 were selected for cDNA synthesis.

RNA (800 ng per sample) was used to generate adaptor-ligated double-stranded cDNA libraries for RNA-Seq using the TruSeq Sample Prep Kit V1 and V2 (Illumina, San Diego, CA) following the manufacturer’s protocol. Quantification and quality inspection of ds-cDNA was carried out using the Experion Automated Electrophoresis System (Bio-Rad, Hercules, CA) and Qubit® 2.0 Fluorometer (Life Technologies, Carlsbad, CA). Samples were diluted to 20 nM and pooled to generate the multiplexed cDNA library (24 adaptor-tagged sample pools total: 3 treatments x 2 time points x 4 replicates).

### Transcriptome sequencing and assembly

The cDNA library (50 fmoles) was sequenced on one flow cell lane using the Illumina HiSeq™2000 platform at the Ohio State University Comprehensive Cancer Center. Four fluorescently labeled nucleotides and a specialized polymerase were used to determine the clusters base by base in parallel. The mean library insert sequence size was 272 bp and both ends of the library were sequenced to generate 100 bp raw paired end reads. The Illumina Analysis Package CASAVA 1.8.2 was used to perform bcl conversion and demultiplexing. Image deconvolution and quality value calculations were carried out using the Illumina GA pipeline v1.6.

Adapter indexes and poly (A) tails were trimmed and duplicates reads removed during the read quality filtering and duplicate read removal stage in the Rnnotator automated pipeline [54] in Galaxy [55] at the OARDC, MCIC. Trimmed reads of low quality (Phred threshold score <20; read length <20 bp) and low complexity (80% of a read with single-nucleotide, di-nucleotide, or tri-nucleotide repeats) were omitted. In addition, low quality reads containing sequence errors (*i.e*. low K-mer sizes) were removed using the K-mer bases approach. Oases v0.2.08 [56] was used for *de novo* assembly with K-mer sizes of 53, 59, 65, 71, 77, 83, and 89. The resulting contigs and singletons were then combined (hereinafter referred to as transcripts). To obtain the set of non-redundant transcripts, reads ≥90% sequence similarity were collapsed into clusters and the longest read retrieved using CD-HIT-EST [57]. Using Minimus2 [58], three independent assemblies were generated (one per treatment) and collapsed into a final assembly that also included transcript sequences from Chen et al. (2012). Assembly parameters are provided in Additional file 6: Table S6. The Illumina sequence reads have been deposited in the NCBI short sequence read archive under the accession number SRP022856.

### Functional annotation and ortholog comparison

Illumina reads that mapped uniquely (i.e., unambiguously) to one transcript in the *de novo* assembled transcriptome were quantified using the CLC Genomics Workbench (v5.1, CLC Bio.). The quantified transcript read files were imported into Bioconductor, an open-source software project based on the R programming language [59]. Using custom R scripts (available on request from BJC), the dataset was filtered to contain only transcripts with a minimum of five mapped reads for any of the three replicates in at least one treatment/time combination. The subset of transcripts meeting this criterion was used in subsequent analyses.

Categorization of *de novo* assembled transcripts was carried out using functional information for orthologs from model organisms*.* Deduced protein sequences were compared to protein sequences in the non-redundant (nr) and Swiss-Prot databases using BLASTx [60] with a significance cut-off *E*-value of <10^-6^. Transcripts with best matches to viral or maize sequences were removed from further analyses. The publicly available platform-independent Java™ 6 implementation of the BLAST2GO software [61] was used for hierarchical classification of gene ontologies (GO terms) on the basis of biological process, molecular function, and cellular component. The top 20 BLAST hits with a cut-off *E*-value of 10^-6^ and similarity cut-off of 55% were considered for GO annotation.

Pair-wise comparisons of transcripts to cDNA databases for eight invertebrate species across five orders were carried out using desktop downloaded tBLASTx software and an *E*-value threshold of 10^-10^. Seven of the invertebrate databases were constructed by retrieving characterized transcriptome sequences (as of June 18, 2012) from the ftp files of NCBI or Ensembl and included: *Acyrthosiphon pisum* (pea aphid, order Hemiptera, 37,994 sequences), *Apis mellifera* (honey bee, order Hymenoptera, 18,542 sequences), *Nasonia vitripennis* (parasitic wasp, order Hymenoptera, 27,287 sequences), *Tribolium castaneum* (red flour beetle, order Coleoptera, 14,366 sequences), *Anopheles gambiae* (malaria mosquito, order Diptera, 14,974 sequences), *Drosophila melanogaster* (fruit fly, order Diptera, 19,233 sequences), and *Caenorhabditis elegans* (soil nematode, order Rhabditida, 32,201 sequences). A NCBI transcript database for *Peregrinus maidis* (maize planthopper, order Hemiptera, 10,636 sequences derived from the gut) was also accessed [22].

### Transcriptome analysis

Using custom R scripts (available on request from BJC), the set of *de novo* assembled transcripts (see above) was normalized by calculating the number of reads per kilobase of exon model per million mapped reads (RPKM) [62]. Quality assessment of the normalized datafile was conducted using the quality control function of transcriptome analysis in CLC Bio. Heat maps for transcriptional profiles were generated using heatmaps.2 in the gplots package in R.

A two-way analysis of variance (ANOVA) model from the nlme package in R was used to assess the impact of two main fixed factors (and their interaction): treatment (feeding on MCDV-infected, MFSV-infected or healthy plants) and time (4 h or 7 d) on transcript accumulation, with replicate being treated as a random factor. Significance for each factor was defined at a *P* value of 0.05 (FDR <0.25 for all transcripts with an ortholog predicted function) for this and all other analyses unless otherwise specified. As time was determined to be a significant factor in the model, *post hoc* statistical tests were carried out for the individual time points using the limma package in R. *Post hoc* tests included Bayes-moderated *t*-tests to identify the set of transcripts differentially expressed among treatments. One-way ANOVA was carried out to determine transcript level differences between *G. nigrifrons* fed on MFSV- and MCDV-infected maize. For comparison purposes differentially expressed transcripts were also identified independently using the Rmodule edgeR [63], which relies on an overdispered Poisson model to moderate the dispersion.

To guide biological interpretation, *G. nigrifrons* transcripts were first assigned a *D. melanogaster* transcript ID, then enrichment of GO and other annotation terms for differentially expressed transcript lists were examined using the DAVID functional annotation tool [25]. The enrichment score assigned to each transcript group (annotation cluster) represents the geometric mean of the EASE Scores (modified Fisher’s Exact Test) associated with each enriched annotation term in the gene group [26,41], which allows for testing the relative importance of the groups rather than a strictly statistical analysis. For this reason, enrichment scores are presented as the minus log transformed geometric mean instead of an absolute *P*-value [26]. Significant clusters were defined as having an enrichment score >1.3 in minus log scale and containing ≥5 transcripts.

### Transcript accumulation profiling using RT-qPCR

Each replication consisted of two treatments, feeding on MFSV-infected or healthy maize, with eight time points, 1 h, 4 h, 1 d, 4 d, 7 d, 2 wk, 3 wk, or 4 wk per treatment. Leafhoppers were collected and stored identically to the RNA-Seq experiment, with the only differences being in time of collection. Each treatment/time point was replicated three times using nymphs from different cohorts.

Total RNA was extracted from pools of 15 leafhoppers, quantified and quality assessed using the protocols outlined above. RNA was digested with DNase I (Invitrogen, Carlsbad, CA) and converted to ss-cDNA using the iScript synthesis kit (Bio-Rad, Hercules, CA). Primer pairs for targeted mRNAs were designed using Primer3 [64] as follows: GnigEST-2650: F-TGCCGAGTTCAAGGCTCTAT and R-CCAGTGTGGCCGATACTTTT; 10779: F-ATGATCGTGGGACTCTCCTG and R-GGACAAAGGCAACGTCAT; 35055: F-GTTCAGAGGTTGGGTCGTGT and R-CTCCACCATCACTCCAAGGT; 3242: F-GTGTCCGTGACTCATGATGG and R-GTGTCCGTGACTCATGATGG. In preliminary experiments, primer concentrations of 50 nM, 300 nM, and 900 nM were tested to determine optimal qPCR conditions for each gene. Elongation factor 1-alpha [23] was used as the endogenous control with forward and reverse primers pairs F - CTACACACCCGTCCTCGATT and R- ACTTGGGGTTGTCCTCAGTG.

The qPCR reactions (15 μl) were performed in duplicate using IQ™ 2X SYBR Green Supermix (Bio-Rad, Hercules, CA) and 300 nM of each primer on a CFX96 Real-Time PCR Detection System according to the manufacturer’s recommendations. Cycling conditions were: 50°C for 2 min; 95°C for 10 min; 41 cycles of denaturation at 95°C for 15 s, and annealing/extension at 60°C for 1 min; and, 95°C for 15 s. PCR efficiency (*E*) was evaluated by performing a dilution series experiment using a target assay and the Equation *E* = 10^(−1/slope)^[65].

Expression levels were measured separately for reference and target genes, using three biological replicates for each treatment/time point combination. Relative transcript abundance was calculated using the $2_{T}^{-\Delta \Delta C}$ method [66]. Threshold cycle (*C*_T_) values reported by the CFX96 Real-Time PCR Detection System were normalized to the reference gene and converted to relative log_2_-fold differences between maize lines and treatments. Two-tailed independent *t*-tests (*P* < 0.05) were used for statistical determination of differential expression between each treatment/time point combination.

### Availability of supporting data

The Illumina sequence data from this study have been submitted as BioProject ID [SUB192597] to the NCBI sequence read archive under the accession [SRP022856]. The Transcriptome Shotgun Assembly project has been deposited at DDBJ/EMBL/GenBank under the accession GAQX00000000.1. The version described in this paper is the first version, GAQX01000000. All the supporting data are included as additional files.

## Competing interests

The authors declare that they have no competing interests.

## Authors’ contributions

BJC, MGR, AM conceived and designed the study. BJC and YP conducted the sampling and did the molecular work (RNA extraction, library preparation). BJC and SW carried out the read filtering, trimming and assembly. BJC and SW performed the differential expression analysis. BJC, LRS, MGR, AM wrote and revised the manuscript. All authors read and approved the final manuscript.

## Supplementary Material

Additional file 1: Table S1 Ortholog amino acid sequence similarity for assembled transcripts. Click here for file

Additional file 2: Table S2 Gene and enzyme ontologies assigned to assembled transcripts using nr database. Click here for file

Additional file 3: Table S3 Transcripts differentially expressed in *G. nigrifons* fed on virus-infected maize for 4 h and 7 d. Click here for file

Additional file 4: Table S4 The 25 top-ranked transcripts with the greatest up-or down-regulation at each time point. Click here for file

Additional file 5: Table S5 The four differentially expressed transcripts from next generation sequencing validated by RT-qPCR. Click here for file

Additional file 6: Table S6 Preprocessing parameters of *de novo* assembly. Click here for file
